# Antifungal Activities and Mode of Action of *Cymbopogon citratus*, *Thymus vulgraris*, and *Origanum heracleoticum* Essential Oil Vapors against *Botrytis cinerea* and Their Potential Application to Control Postharvest Strawberry Gray Mold

**DOI:** 10.3390/foods10102451

**Published:** 2021-10-15

**Authors:** Jiaqi Yan, Hua Wu, Keying Chen, Jiajun Feng, Yansong Zhang

**Affiliations:** 1 College of Horticulture, China Agricultural University, No. 2 Yuanmingyuan West Road, Beijing 100193, China; 13676246788@163.com (K.C.); fengjiajun0323@163.com (J.F.); zhangyansong21@mails.ucas.ac.cn (Y.Z.); 2 Beijing Advanced Innovation Center for Food Nutrition and Human Health, Beijing Technology and Business University, No. 11 Fucheng Road, Beijing 100048, China; wuhua@btbu.edu.cn; 3 Beijing Engineering and Technology Research Center of Food Additives, Beijing Technology and Business University, No. 11 Fucheng Road, Beijing 100048, China; 4 College of Chemistry and Materials Engineering, Beijing Technology and Business University, No. 11 Fucheng Road, Beijing 100048, China

**Keywords:** essential oil, *Botrytis cinerea*, antifungal activity, antifungal mechanism, postharvest quality

## Abstract

Gray mold caused by *Botrytis cinerea* is one of the most destructive postharvest decay of strawberry fruit. The present study aims to identify essential oils with antifungal activity against *B. cinerea* and the underlying mechanisms and their potential application in controlling postharvest decay. In the screening test, essential oils from *Cymbopogon citratus* (*Cc*), *Thymus vulgraris* (*Tv*), and *Origanum heracleoticum* (*Oh*) exhibited maximum inhibition of *B. cinerea* mycelial growth. The three essential oils altered the hyphal morphology and ultrastructure and resulted in many blebs around the hyphae. The essential oils damaged the plasma membrane of *B. cinerea* cells and resulted in the leakage of intercellular nucleic acids, proteins and soluble sugars. The exposure of strawberries to the vapors of these three essential oils in commercial package reduced gray mold, with *Tv* and *Oh* exhibiting strong efficiency and disease index reduction by 53.85% and 57.69%, respectively. *Oh* also inhibited postharvest decay and maintained fruit quality, preventing weight loss and soluble solid degradation. The study proposes using plant essential oils as an alternative to chemical fungicides in controlling the gray mold of strawberries.

## 1. Introduction

Strawberry (*Fragaria* × *ananassa*) is a soft fruit crop ranked first in production among the small berry crops, with a cultivation area of more than 400,000 hectares worldwide and a global production of approximately 9.118 million tons [1]. However, several fungal pathogens attack strawberries due to their tender and juicy characteristics, among which *Botrytis cinerea* is the primary fungal pathogen, causing fruit decay that has resulted in severe economic losses to the strawberry industry [2]. *B. cinerea* is a necrotrophic fungus that attacks the fruits in the field or after harvest and causes gray mold in more than 200 crop species [3]. The fungal conidia are highly abundant and ubiquitous and generally penetrate unripe strawberries through wounds or natural openings and remain quiescent until ripe, causing rapid decay of fruit at or after harvest [4]. Studies have reported from 25% to 55% losses due to *B. cinerea* infection during harvest and up to 89% after harvest [5].

Synthetic fungicides, such as cyprodinil, phenylpyrrole, anilinopyrimidine, and fludioxonil have been used to manage gray mold on strawberries in the field; however, no strategy has been developed for managing postharvest infection [3]. These fungicides, with site-specific action, have been used for more than thirty years, leading to resistant fungal strains. Moreover, the negative impact of fungicide residues on human health and the environment has raised public concerns [6], emphasizing the need for alternative measures to control strawberry gray mold.

The use of natural plant extracts as an alternative to chemical fungicides has gained attention due to their safe and eco-friendly characteristics. Essential oils are volatile aromatic compounds extracted from plant materials by hydrodistillation or pressing processes [7]. The major components in the essential oils responsible for the antifungal properties are terpenes and terpenoids with different functional groups [8]. Research studies have proven that some essential oils could damage the fungal membrane and promote electrolyte leakage, resulting in fungal death. So far, some essential oils have been reported to control plant diseases caused by various fungal pathogens, such as *Botrytis*, *Rhizopus*, *Penicillium*, *Alternaria*, and *Monilinia*, on fresh fruits and vegetables [9,10,11]. However, essential oils to prevent plant disease in the fields are limited due to their volatile characteristics. Meanwhile, the volatile nature of the essential oils also offers an alternative strategy for postharvest handling; the oil can be combined with fruit or vegetable package during postharvest transportation and marketing. A controlled release of essential oils in the package or storage conditions is a safe, effective, and convenient strategy to control postharvest decay, without causing an external bruise [7]. This approach is suitable for soft fruits such as berries that cannot tolerate physical handling during postharvest dipping and coating. In addition, vapor treatment caused little residue on fruit surface, compared to dipping or coating treatment. Our previous study found that mint and thyme essential oils in commercial clamshell reduced *Rhizopus* rot of strawberry and peach fruits [8]. Meanwhile, tea tree essential oil with hot air in storage efficiently controlled the gray mold of strawberries [12]. However, essential oils are abundantly available in nature, but only few have been explored for their antifungal activity and used to control postharvest decay of fresh vegetables and fruits so far. Therefore, it is meaningful to explore more essential oils for their potential application in postharvest decay control.

The present study screened twenty-two essential oils from twelve genera for their antifungal activity against *B. cinerea*, analyzed the major compounds in the essential oils with highly inhibition efficiency, and investigated the mechanism of action of the selected essential oils. Further, the potential application of the selected essential oil vapors in controlling gray mold on artificially inoculated strawberries and the possible impact on fruit quality at room temperature storage were evaluated.

## 2. Materials and Methods

### 2.1. Pathogen Isolate, Essential Oils, and Fruit Materials

The *B. cinerea* isolate ACCC 36058 was procured from the Agricultural Culture Collection of China (ACCC) and grown on potato dextrose agar (PDA) at 25 °C with 12 h of light. Twenty-two essential oils from twelve genera (Table 1) were purchased from Poli Co., Ltd. (Shanghai, China), and stored at 4 °C. Strawberries were harvested from a greenhouse in Changping, Beijing. Fruit at commercial maturity, with uniform size and color and free from physical damage and pathogen infection, were used for the experiments.

### 2.2. Screening of Essential Oils

The essential oil screening was performed as described by Yan et al. [8]. One *B. cinerea* mycelial plug, 5 mm in diameter, was prepared with a sterile iron borer from the edge of a 14-day-old fungal colony and placed in the center of a petri dish (100 mm dia.) containing 20 mL of PDA. Three stainless steel insect pins with a sterile filter paper (6 mm dia.) on top were inserted into the edge of each plate, around the mycelial plug. Essential oil (3, 2 or 1 μL) was added into each filter paper disc to obtain a final volatile concentration of 150, 100, or 50 μL L^−1^. The volatile concentration was calculated by dividing the volume of essential oil on the filter paper disc by the volume of space in the petri dish. Control plates had no essential oil on the filter paper disc. All plates were sealed using parafilm, incubated at 25 °C with 12 h of light, and evaluated after 7 d. The percent inhibition of mycelial growth was calculated using the following formula [8]: Percent inhibition of mycelial growth (%) = [(AC − AE)/AC] × 100%, where AC (cm^2^) is the mean colony area in the control plates and AE (cm^2^) is the mean colony area in the treatment plates. Four replicates were maintained for each essential oil treatment and the experiment was repeated (*n* = 8). The essential oils that exhibited complete inhibition at 150 μL L^−1^ were chosen for the test at 100 μL L^−1^ and those that exhibited complete inhibition at 100 μL L^−1^ were chosen for the test at 50 μL L^−1^.

### 2.3. Analysis of Essential Oil Composition

Essential oils from *Cymbopogon citratus* (*Cc*), *Thymus vulgraris* (*Tv*), and *Origanum heracleoticum* (*Oh*) exhibited maximum *B. cinerea* mycelial growth inhibition in the screening experiment and were selected to analyze the composition and antifungal mechanism. Ten microliter of each essential oil was mixed with 990 μL of n-hexane, and the water residues were removed by using anhydrous sodium sulfate to determine the composition. The essential oils were analyzed by gas chromatography-mass spectrometry (GC-MS; 7890 B Agilent GC equipped with a 5977 A mass selective detector; Agilent Technologies, Santa Clara, CA, USA) with a DB-WAX column (30 m × 320 μm × 0.25 μm); helium gas (99.99%, 3.1 mL min^−1^) was used as carrier gas. The measurements were performed as follows: an initial temperature of 40 °C for 1 min was subsequently elevated to 57 °C at the rate of 5 °C min^−1^, 78 °C at the rate of 2 °C min^−1^, 88 °C at the rate of 4 °C min^−1^, 92 °C at the rate of 3 °C min^−1^, 107 °C at the rate of 1 °C min^−1^, 129 °C at the rate of 6 °C min^−1^, 137 °C at the rate of 4 °C min^−1^, 159 °C at the rate of 6 °C min^−1^, 163 °C at the rate of 4 °C min^−1^, 169 °C at the rate of 1 °C min^−1^, and 230 °C at the rate of 6 °C min^−1^. The injector temperature was 250 °C for the interface and 230 °C for the ion source (70 eV). One microliter of essential oil was injected at a 1:5 split ratio and the volatile compounds were identified by comparing their mass spectra with the existing library (NIST 8) and the retention index (RI) with literatures [13,14].

### 2.4. Analysis of Botrytis cinerea Morphology and Ultrastructure

*B. cinerea* was grown on PDA at 25 °C for 3 days and treated with *Cc*, *Tv*, or *Oh* vapor at a 50 μL L^−1^ concentration for another 24 h. Mycelial plugs (3 mm dia.) were taken from the edge of the fungal colony, fixed with 2.5% (*v*/*v*) glutaraldehyde at 4 °C for 3 h, and washed four times with phosphate buffer (100 mmol L^−1^, pH = 7.2). The plugs were post-fixed with 1% (*v*/*v*) osmium tetroxide at 4 °C for another 2 h and washed three times with phosphate buffer. After dehydration in a series of acetone solutions (30%, 50%, 70%, 80%, and 90%) and thrice in pure acetone for 15 min each, specimens were freeze-dried (Leica EM CPD300; Leica, Wetzlar, Germany), gold-coated (IB-3; Eiko, Hitachinaka, Japan), and observed under a scanning electron microscope (SEM, Model S-3400N; Hitachi, Tokyo, Japan). The remaining specimens were embedded in 1:1 epoxy medium and pure epoxy medium for 12 h each. The ultrathin sections (70 nm in thickness) obtained from these specimens were contrasted with uranyl acetate and lead citrate for 15 min each and observed under a transmission electron microscope (TEM, JEM-1230; Jeol, Tokyo, Japan).

### 2.5. Analysis of Botrytis cinerea Membrane Integrity

*B. cinerea* was grown on PDA at 25 °C for 7 d and at 4 °C under light for 7 d to facilitate sporulation. The conidia were suspended in 5 mL of sterile distilled water containing 0.01% (*v*/*v*) Tween 20 as surfactant and filtered through four layers of sterile cheesecloth to remove the mycelial fragments. Then, 300 μL of the conidial suspension was mixed with 30 mL of diluted potato dextrose broth (5% PDB) and incubated at 25 °C to facilitate mycelial growth. After 7 d of incubation, the mycelia were filtered through four layers of sterile cheesecloth, washed with sterile distilled water, and dissolved in 5 mL of sterile distilled water with 2.5 µL of *Cc*, *Tv*, or *Oh* from the 10 × stock solution (containing 0.1% Tween-20) in a 10 mL centrifuge tube to obtain a final concentration of 50 µL L^−1^. After 6 h of incubation, the mycelia were filtered and washed to analyze the membrane integrity. The filtrates were filtered through a 0.22 μm microporous membrane and used to determine the cytoplasmic leakage. Five replicates were maintained for each essential oil treatment and the experiment was repeated (*n* = 10).

The mycelia were dissolved in 5 mL of sterile distilled water containing 1 mg mL^−1^ of propidium iodide (PI) as a fluorescent indicator and incubated at 4 °C in the dark for 30 min to observe the membrane integrity. After washing with sterile distilled water, the mycelia were placed on slides and observed under a confocal laser scanning microscope (Shimadzu RF-5301 PC, Shimadzu, Kyoto, Japan) at 40 × magnification. The excitation and emission wavelengths were 488 nm and 594 nm, respectively.

The nucleic acid of the filtrates was determined by recording the absorbance at 260 nm with a spectrophotometer (Lambda 35; PerkinElmer Inc., Waltham, MA, USA) and expressed as µg mL^−1^. Soluble protein content of the filtrates was measured using a bicinchoninic acid (BCA) assay and expressed as µg mL^−1^ [15]. The soluble sugar content of the filtrates was determined using the anthrone-sulphuric acid method and expressed as µg mL^−1^ [16].

### 2.6. Effects of Selected Essential Oils on Strawberries Inoculated with Botrytis cinerea

The in vivo evaluation of the selected essential oils on strawberries inoculated with *B. cinerea* was performed following the method of Sun et al. [7] and Zhong et al. [17], with some modification. The conidial suspension was collected as mentioned above and adjusted to 1 × 10^6^ conidia per mL for fruit inoculation. Strawberries were sterilized with 1% hypochlorite acid and wounded (2 mm deep × 2 mm wide) with a sterile nail. Twenty microliters of the conidial suspension was injected into the wound. Six inoculated fruits were placed in a commercial clamshell (133 mm × 109 mm × 90 mm). A filter paper disc (20 mm × 20 mm) with 40 μL of *Cc*, *Tv*, or *Oh* was placed in a Miracloth bag (EMD Millipore Corporation, BillerIca, MA, USA) and attached to the inside of the lid. Then, the fruit were stored at 25 °C with 60% relative humidity (RH). A filter paper disc with 40 μL of water in a Miracloth bag was attached to the lid and maintained as control. The clamshell was not sealed and the essential oil vapor was allowed to disperse during storage. Disease index and disease incidence were evaluated every 12 h. Disease index was determined using a 0–4 scale based on the percentage of lesion area as follows: 0 = 0%, 1 = below 25%, 2 = 25–50%, 3 = 50–75%, and 4 = more than 75% of the total surface. Disease incidence was calculated as the percentage of infected fruit to total fruit. Four replicates were maintained for each essential oil treatment and the experiment was repeated (*n* = 8).

### 2.7. Effects of Selected Essential Oils on Fruit Natural Decayand Postharvest Quality

Six healthy strawberry fruits were placed in a commercial clamshell and treated with *Cc*, *Tv*, or *Oh*, as mentioned above. Natural decay, weight loss, firmness, and soluble solid content (SSC) of the fruits were evaluated every 12 h. The natural decay index was determined based on a 0–4 scale and the natural decay incidence was determined as the percent of decayed fruit to the total fruit, as mentioned above. Fruit weight loss was represented as the percentage of weight loss to the initial weight. Fruit firmness was evaluated using a penetrometer (GY-B, Mudanjiang Mechanical Institute, Heilongjiang, China) equipped with a flat probe (8 mm dia.). Fruit SSC was measured with a digital refractometer (Spectronic Instruments, Rochester, NY, USA) and expressed as % °Brix. Four replicates were maintained for each essential oil treatment, and the experiment was repeated (*n* = 8).

### 2.8. Statistical Analysis

All data were analyzed in the SPSS software (ver. 17.0; Experian QAS, Boston, MA, USA), using the one-way analysis of variance (ANOVA). Ducan’s multiple range test was used to determine the differences in treatment means at α = 0.05.

## 3. Results

### 3.1. Screening of Essential Oils Based on Inhibition Efficiency

Twenty-two essential oils were evaluated for their inhibitory effects on *B. cinerea* mycelial growth using the volatility-based method. Nine essential oils (*Thymus zygis*, *Melaleuca ericifolia*, *Pelargonium graveolens*, *Mentha piperita*, *Thymus satureoides*, *Cymbopogon martini*, *Cymbopogon citratus*, *Thymus vulgraris*, and *Origanum heracleoticum*) at a 150 μL L^−1^ concentration completely inhibited the mycelial growth (100%), while the other essential oils showed an inhibition rate ranging from 10.71% to 97.01%, after 7 d of incubation. Meanwhile, at 100 μL L^−1^, only *Cc*, *Tv*, and *Oh* showed complete mycelial inhibition (100%), with lower efficiency for the other essential oils (from 21.62% to 97.23%). Meanwhile, the inhibition efficiency of *Cc*, *Tv*, and Oh at 50 μL L^−1^ decreased to 72.06%, 98.69%, and 99.32%, respectively (Table 1).

### 3.2. Composition of the Selected Essential Oils

The three essential oils (*Cc*, *Tv*, and *Oh*) that exhibited significant *B. cinerea* inhibition at low concentrations (50 μL L^−1^) were selected to analyze the composition and antifungal mechanism. The GC-MS analysis revealed the complex nature of all three essential oils with more than 30 compounds, primarily including terpenes or terpenoids. In *Cc*, *α*-citral and *β*-citral were the most abundant, representing 38.34% and 29.51% of the total compositions, respectively. Thymol and *p*-cymene were the major components in *Tv* (22.71% and 20.43%, respectively), followed by *γ*-terpinene (11.47%). Carvacrol was the key component in *Oh* (37.47%), followed by *p*-cymene (21.51%; Table 2).

### 3.3. Effects of Cc, Tv, and Oh on Botrytis cinerea Morphology and Ultrastructure

**Figure 1:**
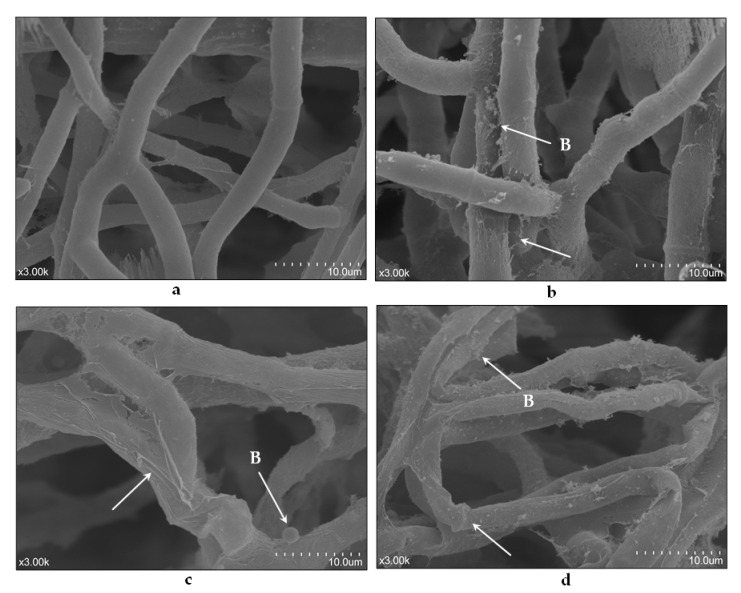
Scanning electron microscopy (SEM) images of *Botrytis cinerea* hyphae exposed to control (**a**), *Cymbopogon citratus* (*Cc*, **b**), *Thymus vulgraris* (*Tv*, **c**), and *Origanum heracleoticum* (*Oh*, **d**) essential oil. Bar = 10.0 µm. B, Belbs.

The changes in *B. cinerea* hyphal morphology under exposure to different essential oils were analyzed under SEM. In control, the normal hyphae had a tubular shape, with a smooth surface (Figure 1a). Meanwhile, treatment with the three essential oils resulted in abnormal hyphae with a rough texture. After exposure to *Cc*, the hyphae exhibited a coarse surface but still had a tubular body (Figure 1b), while *Tv* and *Oh* severely damaged *B. cinerea*, resulting in shrived, distorted, and twisted hyphae; *Oh* exhibited more damage than *Tv* (Figure 1c,d). Besides, treatments with all three essential oils resulted in many blebs, vesicles, and microspheres around the hyphae.

**Figure 2:**
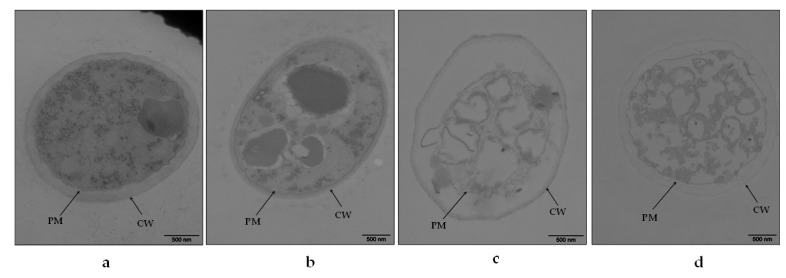
Transmission electron microscopy (TEM) image of *Botrytis cinerea* mycelium exposed to control (**a**), *Cymbopogon citratus* (*Cc*, **b**), *Thymus vulgraris* (*Tv*, **c**), and *Origanum heracleoticum* (*Oh*, **d**) essential oil. Bar = 500 nm. CW, cell wall; PM, plasma membrane.

Furthermore, TEM was used to explore the ultrastructural characteristics of *B. cinerea* treated with different essential oils. The regular cells of the control hyphae had strong cell walls with uniform thickness, smooth plasma membrane close to the cell wall, abundant cytoplasmic matrix, and internal organelles with regular shape and structure (Figure 2a). Exposure to *Cc* altered the ultrastructure, resulting in hyphae with a thinner cell wall but still with a standard shape, abundant matrix, and intact organelles (Figure 2b); *Tv* and *Oh* severely damaged the hyphal cells, exhibiting plasmolysis and cytoplasm lysis (Figure 2c,d).

### 3.4. Effects of Cc, Tv, and Oh on Botrytis cinerea Membrane Integrity

**Figure 3:**
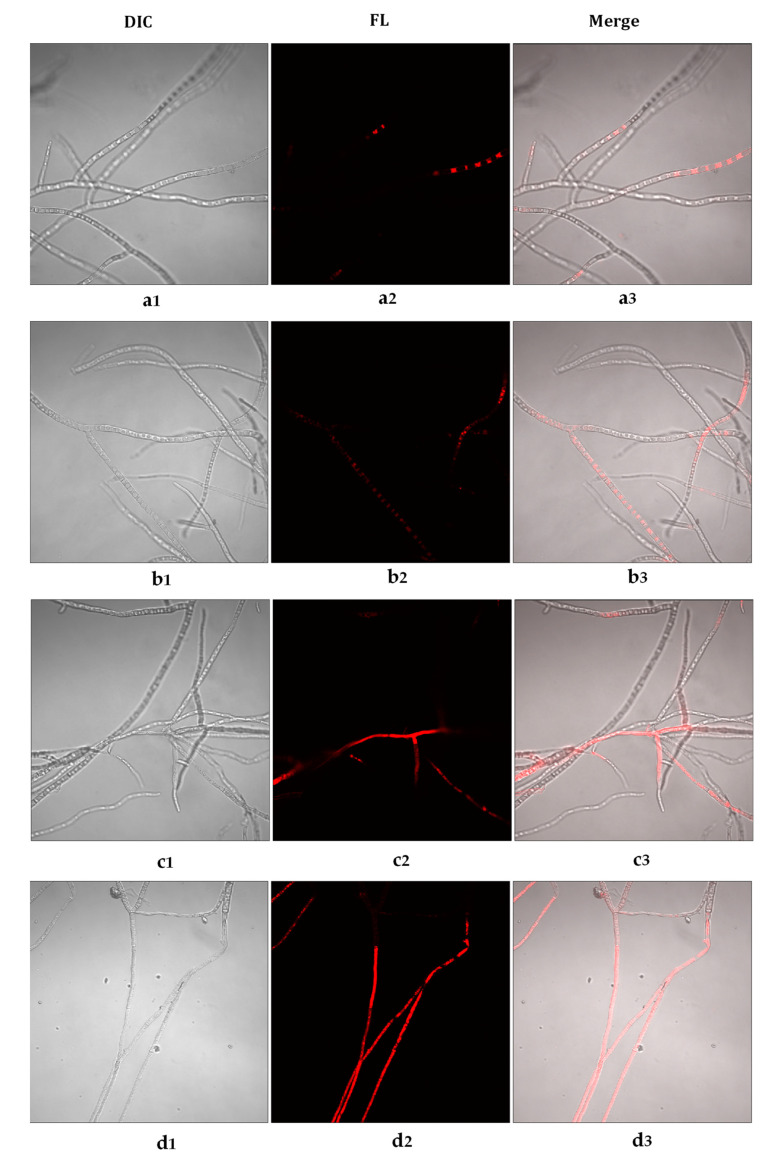
Confocal laser scanning microscopy images of *Botrytis cinerea* mycelium membrane integrity exposed to control (**a1**–**a3**), *Cymbopogon citratus* (*Cc*, **b1**–**b3**), *Thymus vulgraris* (*Tv*, **c1**–**c3**), and *Origanum heracleoticum* (*Oh*, **d1**–**d3**) essential oil. DIC, differential interference contrast images without fluorescence; FL, red fluorescence images with propidiumiodide (PI) combined with nucleic acid.

Propidium iodide (PI) was used to evaluate the membrane integrity of *B. cinerea* hyphae under essential oil exposure. PI is a red fluorescent dye that can combine with a nucleic acid to generate a red fluorescence but cannot penetrate intact plasma membrane. When the plasma membrane gets destroyed, PI enters and develops a red fluorescence visible under a confocal laser scanning microscope. Little fluorescent signal was detected in the control hyphae and the hyphae exposed to *Cc* (Figure 3a1–a3,b1–b3). Meanwhile, hyphae exposed to *Tv* and *Oh* exhibited red fluorescence throughout the hyphae; the hyphae exposed to *Oh* showed more intensive red fluorescence (Figure 3c1–c3,d1–d3).

**Figure 4:**
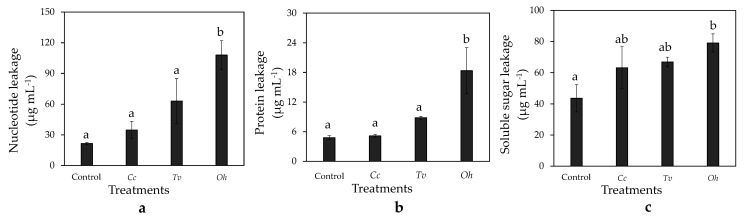
Effects of essential oils from *Cymbopogon citratus* (*Cc*), *Thymus vulgraris* (*Tv*), and *Origanum heracleoticum* (*Oh*) essential oil on leakage of nucleic acids (**a**), proteins (**b**), and soluble sugars (**c**) of *Botrytis cinerea* mycelium. Each value represents the mean of ten replicates. Means with different letters are significantly different based on Ducan’s test (*p* ≤ 0.05).

Leakage of cellular components from *B. cinerea* mycelia was also evaluated to confirm the membrane damage caused by essential oils. The control mycelia exhibited little leakage of nucleic acids, soluble proteins, and soluble sugars. *Cc*, *Tv*, and *Oh* increased the cellular component leakage; however, a significant difference compared with the control was observed only with *Oh* treatment. After 6 h of exposure to *Oh*, a 400.95%, 282.82%, and 81.45% increase in leakage of nucleic acids, soluble proteins, and soluble sugars, respectively, was observed compared with the control (*p* ≤ 0.05; Figure 4a–c).

### 3.5. Effects of Cc, Tv, and Oh on Strawberries Inoculated with Botrytis cinerea

**Figure 5:**
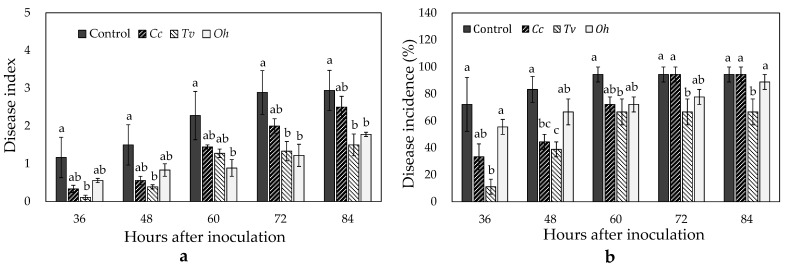
Effects of *Cymbopogon citratus* (*Cc*), *Thymus vulgraris* (*Tv*), and *Origanum heracleoticum* (*Oh*) essential oil vapor treatments on disease index (**a**) and disease incidence (**b**) of strawberry fruit artificially inoculated with *Botrytis cinerea* spore suspension. Each value represents the mean of eight replicates. Means with different letters are significantly different based on Ducan’s test (*p* ≤ 0.05).

Exposure to the three essential oils reduced the disease index (disease severity) on strawberries inoculated with *B. cinerea*; *Tv* and *Oh* exhibited maximum inhibition efficiency (Figure 5a). After 72 h of inoculation and exposure to *Tv* and *Oh*, disease indexes were 53.85% and 57.69%, respectively, lower than that of control (*p* ≤ 0.05). After 84 h of inoculation, *Tv* and *Oh* treatments decreased the disease indexes by 49.06% and 39.62%, respectively, compared with the control (*p* ≤ 0.05).

The three essential oils tended to decrease the disease incidence; however, only *Tv* exhibited continuous and significant inhibitory effects (Figure 5b). After 36, 48, 60, 72, and 84 h of inoculation and exposure to *Tv*, disease incidence was 84.62%, 53.33%, 29.41%, 29.41%, and 29.41% lower than that of control, respectively (*p* ≤ 0.05).

### 3.6. Effects of Cc, Tv, and Oh on Fruit Natural Decay and Postharvest Quality

**Figure 6:**
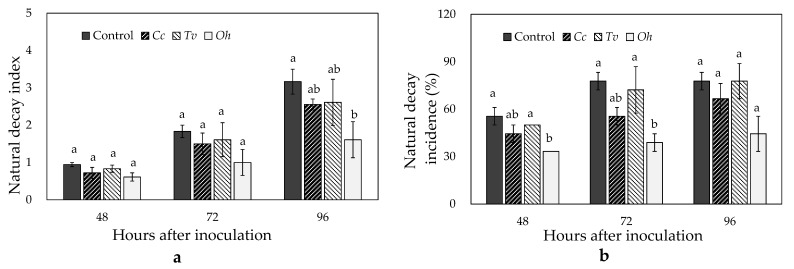
Effects of *Cymbopogon citratus* (*Cc*), *Thymus vulgraris* (*Tv*), and *Origanum heracleoticum* (*Oh*) essential oil vapor treatments on natural decay index (**a**) and natural decay incidence (**b**) of strawberry fruit after harvest. Each value represents the mean of four replicates. Means with different letters are significantly different based on Ducan’s test (*p* ≤ 0.05).

Fruit natural decay happens during storage. In the present study, *Oh* significantly inhibited natural decay at room temperature. After 96 h of storage, *Oh* exposure decreased the natural decay index by 49.2%, compared with the control (*p* ≤ 0.05; Figure 6a). After 24 and 48 h of storage, 55.56% and 77.78% of control fruits developed natural decay, while only 33.33% and 38.89% of *Oh*-exposed fruits developed natural decay (*p* ≤ 0.05; Figure 6b).

Generally, fruit weight loss occurs during storage. Exposure to *Oh* inhibited the fruit weight loss at room temperature. After 12, 24, 36, and 48 h of storage, fruit exposed to *Oh* exhibited 42.21%, 46.32%, 41.63%, and 40.00% less weight loss, respectively, compared with the control fruit (*p* ≤ 0.05; Table 3). Exposure to the three essential oils also maintained fruit SSC during storage. After 48 h of storage, fruit exposed to *Cc*, *Tv*, and *Oh* had 12.29%, 11.04%, and 11.62% more SSC, respectively, than the control (*p* ≤ 0.05; Table 3). None of the three essential oils significantly impacted fruit firmness during storage.

## 4. Discussion

*B. cinerea* is one of the most destructive fungal pathogens of fruit and vegetables that cause severe postharvest decay [2]. The present study screened twenty-two plant essential oils and found that *Cc*, *Tv*, and *Oh* strongly inhibited *B. cinerea* mycelial growth by targeting the hyphal membrane. In vivo experiments proved the inhibitory effect of these three essential oils on the gray mold of strawberry fruit, suggesting these oils as potential alternatives to chemical fungicides in managing postharvest fruit and vegetable decay.

In the screening step, twenty-two essential oils selected from a wide-range of locations and genera (from 11 countries and 12 genera; Table 1) were tested. The essential oils exhibited a wide-range of antifungal efficiency, depending on the source genera and volatile concentrations. Among the tested essential oils, those from *Pinus*, *Artemisia*, *Rosmarinus*, *Ocimum*, *Lavandula*, and *Syzygium* exhibited little activity against *B. cinerea*, even at a relatively high volatile concentration (150 μL L^−1^). A previous study also reported low inhibitory efficiency of *Pinus* and *Rosmarinus* essential oils against *R. stolonifer* [8], indicating their unsuitability as a microbiological inhibitor for postharvest decay control. The essential oils from *Melaleuca*, *Cymbopogon*, and *Mentha* showed a wide-range of inhibitory activity against *B. cinerea*, depending on their species, while those from *Thymus*, *Pelargonium*, and *Origanum* exhibited intense inhibitory activity against *B. cinerea*, even at a relatively low concentration (50 μL L^−1^). *Thymus* essential oil also showed remarkable inhibitory efficiency against *R. stolonifera*, previously [8], suggesting its high application potential in controlling postharvest pathogens. However, little has been reported about *Pelargonium* and *Origanum*. Among those essential oils, *Cymbopogon citratus* (*Cc*), *Thymus vulgraris* (*Tv*), and *Origanum heracleoticum* (*Oh*) exhibited the strongest inhibition efficiency.

Generally, plant essential oils contain several components at different concentrations, with only two or three components representing from 70% to 80% of the total oil composition. These major components contribute to the antifungal activity of the oil [18]. In the present study, *Oh* exhibited the most substantial inhibition against *B. cinerea*, followed by *Tv* and *Cc*. The GC-MS analysis revealed differences in the composition and major components among these oils, probably contributing to the differences in inhibitory efficiency. In *Oh*, carvacrol was the main component (37.47%), followed by *p*-cymene and *γ*-terpinene. Carvacrol is a monoterpene with a phenolic ring derived from *p*-cymene and *γ*-terpinene; both *p*-cymene and *γ*-terpinene are hydrocarbon monoterpenes [19]. Usually, it is terpene, or terpenoid, the most abundant component of essential oils, that contributes to antifungal activity. Monoterpenes with functional structures contribute to higher antifungal activity than the hydrocarbon monoterpenes; the ester, ketonic, aldehyde, alcoholic, and phenolic structures possess from low to high activity, respectively [20]. Therefore, these components, especially carvacrol, contributed to the high inhibitory efficiency of *Oh* against *B. cinerea*.

Meanwhile, in *Tv*, thymol was the main component (22.71%), followed by *p*-cymene and *γ*-terpinene. Thymol is an isomer of carvacrol, with differences in the hydroxyl group position [11]. Studies have reported that the antifungal activity of thymol and carvacrol was related to the hydroxyl group position and the species of fungus. Numpaque et al. [21] found that thymol and carvacrol exhibited a relatively high antifungal activity against *Colletotrichum acutatum* and *Botryodiplodia theobtomae*, with slightly higher activity for thymol. Meanwhile, Kim et al. [22] found thymol and carvacrol could inhibit *Phytophthora cactorum* and *Cryponectria parasitic*; however, carvacrol was more effective than thymol. The lower *B. cinerea* inhibition efficiency of *Tv* than that of *Oh* was probably due to the difference in the hydroxyl group position in carvacrol and thymol or the relative lower concentration of thymol in *Tv*, which needs further study.

Unlike *Oh* and *Tv*, *Cc* had citral as the main component. Citral is an acyclic *α*, *β*-unsaturated monoterpene aldehyde, consisting of the cis-isomer geranial (*α*-citral) and the trans-isomer neral (*β*-Citral) [23,24]. In *Cc*, *α*-citral and *β*-citral represented 38.34% and 29.51% of the total oil composition. Studies have reported the broad-spectrum inhibitory effects of citral against various postharvest fungal pathogens, including *Aspergillus*, *Geotrichum*, and *Penicillium* [23,25]. In the present study, *Cc* exhibited relatively higher inhibition efficiency against *B. cinerea* than the other essential oils, primarily due to citral. However, citral is a monoterpene with aldehyde structure; thus, *Cc* exhibited relatively lower antifungal activity than *Tv* and *Oh* containing thymol and carvacrol with a phenolic structure.

All three essential oils caused hyphal morphology deformation and cell structure alternation in the present study, with *Oh* and *Tv* afflicting the most severe damages, consistently with their high antifungal activities. Besides, many blebs, vesicles, and microspheres were found around the hyphae exposed to the essential oils, indicating apoptosis (programmed cell death) as a part of the antifungal mechanism. In fungi, programmed cell death is a self-destructive process that occurs naturally during reproduction and aging. Environmental stress and toxic metabolites also induce programmed cell death [26]. During apoptosis, the cell cytoskeleton breaks up and the cell membrane bulges outward, forming tiny surface blebs that enlarge into dynamic blebs, carrying organelle fragments [27]. In this study, tiny surface blebs were observed around the hyphae exposed to *Oh* and *Tv* and large dynamic blebs were seen around *Cc*-treated fungal cells. These observations clearly indicate that the essential oils caused apoptosis, similar to caspofungin and plantaricin peptides that killed *Candida albicans* by causing cellular apoptosis [28,29]. Similarly, the antifungal peptide P852 caused bleb formation around *Fusarium oxysporum* hyphae and killed it [30].

The distortion of *B. cinerea* hyphae observed in this study was related to the disrupted cell membrane. Studies have identified membrane-bound enzymes on the fungal plasma membrane that regulate the synthesis of cell wall polysaccharide components. Any minor changes in the cell membrane may affect its function, causing hyphal distortion [6]. In this study, *Cc*, *Tv*, and *Oh* damaged *B. cinerea* plasma membrane, with *Tv* and *Oh* exhibiting severe effects, consistent with hyphal deformation levels. Membrane damage leads to the leakage of intracellular components to the extracellular matrix [6]. In this study, *Oh* significantly increased the leakage of intracellular nucleic acids, proteins, and soluble sugars into the extracellular matrix; PI staining confirmed the most severe damage on *B. cinerea* was after *Oh* exposure.

Therefore, it should be the citral, carvacrol, and thymol in these three essential oils that bounded to the plasma membrane and damaged its permeability. All three compounds are monoterpenes with hydroponic and lipophilic characteristics, making it easy to attach to the membrane’s hydrophobic region and dissolve in the phospholipid bilayer, leading to fungal death [8]. In carvacrol and thymol, the phenolic hydroxyl groups might have damaged the cell membrane by oxidative phosphorylation uncoupling, which contributed to the higher antifungal activities of *Tv* and *Oh* [11].

The major advantage of using essential oils during postharvest transportation and marketing is their volatile feature; they can be combined with the fruit package and released during storage, transportation, and marketing, without any residue or taste problem. Vapor-based treatment is a good option for delicate fruits such as berries that cannot bear intensive postharvest handling. In the present study, the selected essential oils, *Cc*, *Tv*, and *Oh*, were combined with strawberry commercial clamshell to evaluate its potential application on postharvest strawberry decay control. *Tv* and *Oh* exhibited higher disease control efficiency in strawberry inoculated with *B. cinerea* than *Cc*, consistent with their in vitro antifungal efficiency. Studies also reported that the combination of modified atmosphere packaging with essential oils significantly reduced postharvest decay on blueberries, cherry tomatoes, lychees, and grapes [7,31].

Besides *B. cinerea*, strawberry fruit also bears other fungal pathogen infections, such as *Rhizopus* spp., *Mucor* spp., *Colletotrichum* spp., and *Penicillium* spp. [32]. In this study, we evaluated the inhibitory efficiency of *Cc*, *Tv*, and *Oh* on postharvest natural decay, and *Oh* exhibited strong efficiency, indicating that *Oh* might have high antifungal activity against *Rhizopus* spp., *Mucor* spp., *Colletotrichum* spp., or *Penicillium* spp. The main component in *Oh*, carvacrol, is a broad-spectrum antifungal agent and has been reported to inhibit a lot of fungal pathogens in vitro [7]. However, there is no report about *Oh* antifungal activity and further research is needed.

During storage at room temperature, strawberry fruit suffers weight loss, softening, and SSC degradation. The present study found that *Oh* exposure prevented fruit weight loss through the whole storage and tended to maintain fruit firmness. It is reported that carvacrol, the main compound in *Oh*, could inhibit fungal and bacterial growth on blueberry surface, thus maintaining the fruit cuticle integrity, which would prevent fruit from water evaporation and weight loss, subsequently maintaining fruit turbulence and firmness [7]. In the present study, *Oh* exhibited a broad-spectrum antifungal activity to control strawberry natural decay, subsequently preventing fruit weight loss and softening. All the three essential oils also maintained fruit SSC degradation in this study. It was proposed that the essential oils reduced fruit metabolism, which was in accordance with the previous study on mandarins [33].

## 5. Conclusions

To conclude, the present study identified three essential oils, *Cc*, *Tv*, and *Oh*, with intense antifungal activity against *B. cinerea* mycelial growth through a membrane-targeted mechanism. They could disrupt plasma membrane integrity, alter hyphal morphology, and promote apoptosis. In addition, all three essential oils incorporated in commercial clamshell inhibited postharvest gray mold on strawberry fruit, with *Tv* and *Oh* exhibiting strong efficiency. *Oh* exposure also prevented strawberry natural decay and maintained fruit quality.

## Figures and Tables

**Table 1:** Percent inhibition of mycelial growth of *Botrytis cinerea* by essential oil vapor.

		Essential Oil Concentrations		
Essential Oil	Origin	150 µL L^−1^	100 µL L^−1^	50 µL L^−1^
*Pinus sylvestris*	Bosnia	10.71 ± 3.61 a	--	--
*Artemisia indica*	Nepal	43.21 ± 2.95 b	--	--
*Pinus nigra*	Bosnia	60.70 ± 5.97 c	--	--
*Melaleuca viridiflora*	Madagascar	64.41 ± 9.04 cd	--	--
*Melaleuca leucadendron*	Indonesia	70.96 ± 13.85 d	--	--
*Rosmarinus officinalis*	Spain	72.30 ± 1.70 de	--	--
*Cymbopogon khasans*	India	73.41 ± 1.97 de	--	--
*Ocimum sanctum*	India	81.55 ± 0.47 ef	--	--
*Lavandula angustifolia*	Bulgaria	81.74 ± 3.07 ef	--	--
*Ocimum basilicum*	Egypt	82.53 ± 3.67 ef	--	--
*Lavandula latifolia*	Spain	85.92 ± 0.20 f	--	--
*Syzygium aromaticum*	Indonesia	86.78 ± 1.35 f	--	--
*Mentha spicata*	India	97.01 ± 0.66 g	--	--
*Thymus zygis*	Spain	100 ± 0.00 g	21.62 ± 2.72 a	--
*Melaleuca ericifolia*	Australia	100 ± 0.00 g	74.42 ± 4.52 b	--
*Pelargonium graveolens*	Morocco	100 ± 0.00 g	84.85 ± 5.12 c	--
*Mentha piperita*	India	100 ± 0.00 g	85.84 ± 4.16 c	--
*Thymus satureoides*	Spain	100 ± 0.00 g	89.62 ± 1.39 cd	--
*Cymbopogon martinii*	India	100 ± 0.00 g	97.23 ± 2.77 de	--
*Cymbopogon citratus* (*Cc*)	India	100 ± 0.00 g	100 ± 0.00 e	72.06 ± 6.47 a
*Thymus vulgraris* (*Tv*)	Spain	100 ± 0.00 g	100 ± 0.00 e	98.69 ± 0.33 b
*Origanum heracleoticum* (*Oh*)	France	100 ± 0.00 g	100 ± 0.00 e	99.32 ± 0.19 b

Each value is the mean ± standard error of 8 replicates. Within a column, means followed by different letters are significantly different based on Ducan’s test (*p* ≤ 0.05).

**Table 2:** Chemical composition of essential oils from *Cymbopogon citratus* (*Cc*), *Thymus vulgraris* (*Tv*), and *Origanum heracleoticum* (*Oh*).

Compounds	Retention Index (RI) ^a^	Retention Index (RI) ^b^	Percentage (%) ^c^		
			*Origanum heracleoticum*	*Thymus vulgraris*	*Cymbopogon citratus*
α-Thujene	1025	1023	0.74	0.57	--
α-Pinene	1037	1026	1.69	1.59	--
Camphene	1081	1071	0.56	2.25	2.06
β-Pinene	1122	1114	0.29	1.97	0.2
3-Carene	1135	1130	0.19	--	--
β-Myrcene	1152	1164	3.34	--	--
L-Limonene	1181	1196	0.55	0.69	2.92
β-Phellandrene	1214	1164	0.40	0.27	--
Eucalyptol	1216	1211	--	2.95	--
**γ-Terpinene**	1240	1230	7.80	**11.47**	--
trans-β-Ocimene	1242	1245	0.33	--	--
***p*-Cymene**	1265	1273	**21.51**	**20.43**	--
Terpinolene	1275	1270	2.15	2.47	--
Linalool	1542	1540	1.2	5.81	3.52
(+)-2-Bornanone	1544	1539	--	4.05	--
cis-Verbenol	1546	1551	--	--	0.64
α-Caryophyllene	1577	1593	0.34	0.14	--
Terpinen-4-ol	1595	1584	--	2.65	--
β-Caryophyllene	1599	1565	2.6	3.11	2.27
Bornyl acetate	1603	1595	--	0.31	--
Menthol	1630	1626	0.39	--	--
4-Terpinenyl acetate	1631	1613	0.32	--	--
trans-Dihydrocarvone	1632	1642	0.22	--	--
Levomenthol	1633	1626	0.21	0.14	--
Alloaromadendrene	1643	1639	--	0.16	--
cis-β-Farnesene	1654	1648	0.21	--	--
Borneol	1680	1705	2.09	6.11	--
γ-Muurolene	1683	1681	0.32	--	--
Geraniol formate	1689	1667	--	--	0.62
α-Terpineol	1691	1680	2.23	1.43	2.36
**β-Citral**	1694	1689	--	--	**29.51**
Nerol acetate	1698	1700	0.20	--	--
β-Bisabolene	1700	1717	3.03	--	--
Verbenone	1701	1699	--	0.17	--
**α-Citral**	1742	1737	--	--	**38.34**
δ-Cadinene	1753	1757	0.6	0.44	--
γ-Cadinene	1756	1766	0.28	0.23	0.35
Geraniol acetate	1760	1770	0.43	--	6.36
cis-Carveol	1850	1848	--	--	0.17
Carvacryl acetate	1896	1890	0.25	--	--
cis-Geraniol	1900	1884	--	--	0.75
Lemonol	1956	1921	--	--	6.41
Caryophyllene oxide	1967	2002	2.47	0.80	1.75
Epiglobulol	2001	2000	--	0.54	--
Spathulenol	2133	2129	--	0.32	--
**Thymol**	2159	2152	5.32	**22.71**	--
16-Kaurene	2162	2167	--	--	0.44
**Carvacrol**	2166	2161	**37.47**	6.22	1.32
β-Guaiene	2170	2155	0.26	--	--

Boldness indicates the main compounds in each essential oil. ^a^ Retention index, DB-WAX polar column relative to C8-C24 n-alkanes. ^b^ Retention index from literature. ^c^ Relative percentage obtained from peak area.

**Table 3:** Effects of *Cymbopogon citratus* (*Cc*), *Thymus vulgraris* (*Tv*), and *Origanum heracleoticum* (*Oh*) essential oil vapor treatments on weight loss, firmness, and solid soluble content (SSC) of strawberry fruit.

Storage Day	Treatment	Weight Loss (%)	Firmness (g)	SSC
**0**	**Control**	0.00 ± 0.00	68.3 ± 5.51	13.03 ± 0.07
**12**	**Control**	1.54 ± 0.19 a	68.13 ± 6.93 a	10.70 ± 0.10 a
	***Cc***	1.40 ± 0.30 a	75.57 ± 5.87 a	11.20 ± 0.32 ab
	***Tv***	1.54 ± 0.19 a	76.48 ± 8.54 a	11.53 ± 0.09 bc
	***Ov***	0.89 ± 0.43 b	69.78 ± 3.05 a	11.83 ± 0.07 c
**24**	**Control**	3.26 ± 0.24 a	63.47 ± 4.61 a	10.87 ± 0.23 a
	***Cc***	2.91 ± 0.29 a	72.85 ± 4.93 a	11.03 ± 0.47 a
	***Tv***	2.93 ± 0.22 a	73.42 ± 6.38 a	12.27 ± 0.12 b
	***Ov***	1.75 ± 0.26 b	71.26 ± 3.50 a	13.13 ± 0.03 b
**36**	**Control**	4.66 ± 0.36 a	60.93 ± 3.86 a	11.87 ± 0.13 a
	***Cc***	4.37 ± 0.35 a	66.53 ± 7.72 a	11.80 ± 0.12 a
	***Tv***	4.60 ± 0.21 a	66.50 ± 3.09 a	12.53 ± 0.15 b
	***Ov***	2.72 ± 0.22 b	65.97 ± 2.62 a	12.23 ± 0.28 ab
**48**	**Control**	5.95 ± 0.49 a	60.78 ± 3.85 a	10.33 ± 0.12 a
	***Cc***	5.54 ± 0.42 a	64.47 ± 8.03 a	11.60 ± 0.12 b
	***Tv***	5.95 ± 0.19 a	62.58 ± 5.11 a	11.47 ± 0.63 b
	***Ov***	3.57 ± 0.12 b	66.37 ± 4.35 a	11.53 ± 0.17 b

Each value is the mean ± standard error of 8 replicates. Within a column on the same day, means followed by different letters are significantly different based on Ducan’s test (*p* ≤ 0.05).
